# Differences in Functional Brain Connectivity Alterations Associated with Cerebral Amyloid Deposition in Amnestic Mild Cognitive Impairment

**DOI:** 10.3389/fnagi.2015.00015

**Published:** 2015-02-19

**Authors:** Dahyun Yi, Young Min Choe, Min Soo Byun, Bo Kyung Sohn, Eun Hyun Seo, Jiyoung Han, Jinsick Park, Jong Inn Woo, Dong Young Lee

**Affiliations:** ^1^Department of Neuropsychiatry, Clinical Research Institute, Seoul National University Hospital, Seoul, South Korea; ^2^Department of Neuropsychiatry, Seoul Metropolitan Boramae Medical Center, Seoul, South Korea; ^3^Division of Natural Medical Sciences, College of Health Science, Chosun University, Gwangju, South Korea; ^4^Department of Biomedical Engineering, Hanyang University, Seoul, South Korea

**Keywords:** amnestic mild cognitive impairment, amyloid-beta deposition, brain functional connectivity, default mode network, salience network

## Abstract

Despite potential implications for the early detection of impending Alzheimer’s disease (AD), very little is known about the differences of large-scale brain networks between amnestic mild cognitive impairment (aMCI) with high cerebral amyloid-beta protein (Aβ) deposition (i.e., aMCI+) and aMCI with no or very little Aβ deposition (i.e., aMCI−). We first aimed to extend the current literature on altering intrinsic functional connectivity (FC) of the default mode network (DMN) and salience network (SN) from cognitively normal (CN) to AD dementia. Second, we further examined the differences of the DMN and the SN between aMCI−, aMCI+, and CN. Forty-three older adult (12 CN, 10 aMCI+, 10 aMCI−, and 11 AD dementia) subjects were included. All participants received comprehensive clinical and neuropsychological assessment, resting-state functional magnetic resonance imaging, structural MRI, and Pittsburgh compound-B-PET scans. FC data were preprocessed using multivariate exploratory linear optimized decomposition into independent components of FMRIB’s Software Library. Group comparisons were carried out using the “dual-regression” approach. In addition, to verify presence of gray matter volume changes with intrinsic functional network alterations, voxel-based morphometry was performed on the acquired T1-weighted data. As expected, AD dementia participants exhibited decreased FC in the DMN compared to CN (particularly in the precuneus and cingulate gyrus). The degree of alteration in the DMN in aMCI+ compared to CN was intermediate to that of AD. In contrast, aMCI− exhibited increased FC in the DMN compared to CN (primarily in the precuneus) as well as aMCI+. In terms of the SN, aMCI− exhibited decreased FC compared to both CN and aMCI+ particularly in the inferior frontal gyrus. FC within the SN in aMCI+ and AD did not differ from CN. Compared to CN, aMCI− showed atrophy in bilateral superior temporal gyri whereas aMCI+ showed atrophy in right precuneus. The results indicate that despite the similarity in cross-sectional cognitive features, aMCI− has quite different functional brain connectivity compared to aMCI+.

## Introduction

Mild cognitive impairment (MCI) refers to the clinical state of cognitive decline that is greater than expected for a given age and educational attainment but does not interfere with the activities of daily living. In general, MCI is considered as a transitional stage or an intermediate state between normal aging and dementia. Particularly, amnestic MCI (aMCI) has been considered as a prodromal stage of Alzheimer’s disease (AD) dementia (Morris, 2006). Among aMCI individuals, however, nearly half does not show abnormal levels of cerebral amyloid-beta (Aβ) accumulation, which is considered as the hallmark of AD (Price et al., 2005; Rowe et al., 2007; Wolk et al., 2009; Nordberg et al., 2013).

Given that Aβ cerebral deposition is considered as a necessary pathological process of AD (Hardy and Selkoe, 2002; Villemagne et al., 2008), aMCI with high levels of Aβ deposition (aMCI+) may more specifically be the prodromal state of AD dementia compared to aMCI with low levels of Aβ deposition (aMCI−), which may be associated with pathophysiological processes other than AD. However, only a few studies investigated the differences in the clinical or neuroimaging characteristics of aMCI+ and aMCI−. Characterizing the differences between aMCI+ and aMCI− is anticipated to shed light on the underlying mechanisms of each state more specifically.

In the past several years, converging pieces of evidence from structural and functional magnetic resonance imaging (MRI) studies suggested that AD affects specific large-scale brain networks. Particularly, the studies using resting-state functional magnetic resonance imaging (rs-fMRI) – an imaging method that measures functional connectivity (FC, i.e., synchronous ongoing brain activity) between spatially distinct brain regions – have shown that individuals with an early stage of AD dementia or aMCI have disruptions in FC between the structures that are parts of the network referred to as the default mode network (DMN) (Biswal et al., 1995; Raichle et al., 2001; De Luca et al., 2006; Wang et al., 2006; Fox and Raichle, 2007; Sorg et al., 2007; He et al., 2009; Smith et al., 2009; Scholvinck et al., 2010). The DMN, which is comprised of a set of brain regions that are active during rest and deactivated when engaged in cognitively demanding tasks, also shows a striking overlap with the brain regions with high Aβ deposition in AD (Buckner et al., 2005; Hedden et al., 2009; Sperling et al., 2009; Sheline et al., 2010).

In addition to the DMN, abnormal activity within the salience network (SN) – comprised of paralimbic structures such as insula and anterior cingulate cortex – is implicated as another indicator of different neurodegenerative diseases, such as frontotemporal dementia (FTD) (Zhou et al., 2010; Farb et al., 2013). Furthermore, the interaction between the DMN and the SN is thought to be important in generating controlled behavior, particularly for the role of the SN in disengaging the DMN when on a task (Rilling et al., 2008; Sridharan et al., 2008; Sharp et al., 2010; Bonnelle et al., 2012).

Despite a growing literature on disruptions of the DMN and the SN in AD and other neurodegenerative conditions, little is known about differential alterations of the DMN or the SN between aMCI+ and aMCI−. In this context, we first aimed to expand current literature on changes in connectivity strengths of the DMN and the SN from cognitively normal (CN) to AD process by specifically including the analysis of aMCI+ rather than overall MCI. Second, we examined the FC differences in the DMN and the SN between aMCI+ and aMCI− by comparing them to CN as well as to each other.

## Materials and Methods

### Participants

Forty-three older adults (12 CN, 20 aMCI, and 11 AD) were recruited from a dementia clinic of the Seoul National University Hospital. CN subjects did not have subjective or reported cognitive complaints, had Clinical Dementia Rating (CDR) score of 0 (Morris, 1993) and the Mini-Mental State Examination (MMSE) score greater than or equal to 26 (Lee et al., 2002), and performed within the normal range on comprehensive neuropsychological assessment. Individuals with aMCI met Petersen’s criteria (Petersen, 2004): (a) memory complaint corroborated by an informant; (b) objective memory impairment for age, education, and gender; (c) essentially preserved general cognitive function; (d) largely intact functional activities; and (e) not demented. All aMCI individuals had an overall CDR (Morris, 1993) of 0.5. In terms of the criterion (b), a performance score for at least one of the four episodic memory tests included in the Consortium to Establish a Registry for Alzheimer’s disease (CERAD) neuropsychological battery [namely, Word List Memory (WLM), Word List Recall (WLR), Word List Recognition (WLRc), and Constructional Recall (CR) test] (Morris et al., 1989; Lee et al., 2002) was at least 1.5 SD below the respective age-, education-, and gender-specific normative mean (Lee et al., 2004). Patients diagnosed with AD dementia met the National Institute of Neurological and Communicative Diseases and Stroke/Alzheimer’s Disease and Related Disorders Association (NINCDS-ADRDA) criteria for the probable AD (McKhann et al., 1984).

The exclusion criteria for all subjects were: (a) any present serious medical, psychiatric, and neurological disorders that could affect the mental function; (b) evidence of focal brain lesions on MRI; (c) the presence of severe behavioral or communication problems that would make a clinical examination or brain scans difficult; (d) left-handedness; (e) absence of a reliable informant; and (f) illiteracy. Individuals with minor physical abnormalities (e.g., diabetes with no serious complications, essential hypertension, and mild hearing loss) were included. The Institutional Review Board of the Seoul National University Hospital, South Korea, approved the study, and subjects or their legal representatives gave written informed consent.

### Clinical and neuropsychological assessment

All participants were administered a standardized clinical assessment according to the protocol of the Korean version of the CERAD Assessment Packet (Lee et al., 2002). The CERAD neuropsychological battery and the Stroop Test (Seo et al., 2008) were administered by experienced clinical neuropsychologists. Reliable informants provided information regarding participants’ cognitive, emotional, and functional changes as well as medical history. A panel of psychiatrists and a clinical neuropsychologist with expertise in dementia research made clinical decisions on the diagnoses.

### PiB-PET image acquisition and preprocessing

Participants underwent Carbon-11-labeled PiB (^11^C-PiB) PET imaging using the ECAT EXACT 47 scanner (Siemens-CTI, Knoxville, TN, USA), which has an intrinsic resolution of 5.2-mm full width at half maximum (FWHM) and the images of 47 contiguous transverse planes with a 3.4-mm thickness for a longitudinal field of view of 16.2 cm. Before administering ^11^C-PiB, a 10-min transmission scanning was performed using rotating three germanium-68 rod sources to correct the attenuation. Sixty minutes after the intravenous injection of 370 MBq ^11^C-PiB, three 10-min frames of data acquisition were started and later summed into a single frame (60–90 min). All the data were reconstructed in 123 × 128 × 47 matrix with a pixel size of 2.57 mm × 2.57 mm × 3.75 mm using the filtered back projection method with Shepp–Logan filter (cutoff = 0.35 cycle/pixel), and reconstructed images were corrected for attenuation and rearranged onto transaxial, sagittal, and coronal images.

The details of PiB quantification image analyses were described previously (Choo et al., 2011). Briefly, image preprocessing for statistical analyses was performed using SPM2 implemented in MatLab. ^11^C-PiB-PET data of each subject were co-registered to individual volumetric magnetic resonance image and then automatically spatially normalized into the standard MNI template in SPM2 using transformation parameters derived from the normalization of individual magnetic resonance image to the template. All normalized images were reformatted with a voxel size of 2 mm × 2 mm × 2 mm. For quantitative normalization of cerebral ^11^C-PiB uptake values, the cerebellum was used as a reference region (Lopresti et al., 2005) and ^11^C-PiB retention maps as region-to-cerebellar ratio were generated by dividing regional uptake values by the individual mean cerebellar uptake values in the same images. The automatic anatomic labeling algorithm (Tzourio-Mazoyer et al., 2002) and a region combining method (Reiman et al., 2009) were applied to set regions of interest (ROIs) to characterize ^11^C-PiB retention level in frontal, lateral parietal, posterior cingulate–precuneus (PC–PRC), lateral temporal, and basal ganglia (BG) regions. A global cortical ROI consisting of frontal, lateral parietal, PC–PRC, lateral temporal, and BG ROIs was also defined. For each ROI, mean value was calculated by averaging ^11^C-PiB retention values for all voxels within the ROI. Each aMCI participant was classified as PiB-positive (i.e., aMCI+) if ^11^C-PiB retention value of the image was over 1.4 in one of the five ROIs (i.e., frontal, lateral temporal, lateral parietal, PC–PRC, and BG) and PiB-negative (i.e., aMCI−) if ^11^C-PiB retention values of all of the ROIs were equal to or less than 1.4 (Reiman et al., 2009).

### MRI acquisition

Imaging was performed on a 3.0-T GE whole body imaging system (GE VH/I; General Electric, Milwaukee, WI, USA). The rs-fMRI BOLD data for each participant consisted of 100 T2*-weighted single-shot gradient echo EPI sequence with the following parameters: TR/TE/FA = 3000 ms/30 ms/90^o^; voxel size, 1.87 mm × 1.87 mm × 5.0 mm; 26 anterior commissure–posterior commissure aligned axial slices in interleaved order; matrix 128 × 128; scan time ~9 min. During the whole functional scanning, all participants were asked to keep their eyes closed, to stay awake during the entire session, and not to focus their minds on anything in particular. Cushions and headphones were used to reduce subject motion and scanner noise. For structural imaging, we obtained a three-dimensional T1-weighted spoiled gradient recalled echo (SPGR) sequence (TR = 22.0 ms, TE = 4.0 ms, slice thickness/gap = 1.40 mm, matrix = 256 × 192, FOV = 240 mm, Flip angle = 40^o^).

### Data processing and analyses

Data analyses were carried out using multivariate exploratory linear optimized decomposition into independent components (MELODIC) of FMRIB’s Software Library (FSL version 4.0.4)^1^ to identify large-scale patterns of temporal signal-intensity coherence, interpreted as FC, in the population of subjects (Beckmann et al., 2005). Preprocessing included discarding the first five volumes to let the scanner reach equilibrium due to progressive saturation, motion correction, removal of non-brain structures, spatial smoothing (Gaussian kernel of 5-mm FWHM), slice timing correction, and high-pass temporal filtering (100 s). The rs-fMRI volumes were registered to Montreal Neurological Institute-152 standard space (MNI-152). Then, each subject’s preprocessed functional data were run through single-session independent component analysis (ICA) of MELODIC to identify artifacts to be denoised. Finally, the denoised functional data were temporally concatenated across all subjects (covering each comparisons) to create a single 4D group ICA (gICA) data set for the following analysis. The sample-specific DMN and SN were identified from the gICA results.

The between-subject analyses were carried out using the “dual-regression” approach, allowing for voxel-wise comparisons of resting FC (Filippini et al., 2009). In summary, dual-regression included the following steps: (1) obtaining matrices describing the temporal dynamics for each component and subject resulting from using the gICA spatial maps in a linear model fit against the separate rs-fMRI data set, which results in single-subject time-courses corresponding to each of spatial components generated by the gICA over all subjects; (2) normalizing the temporal modes to unit variance; (3) using the set of normalized individual temporal modes as regressors in a first-level GLM to derive individual subject spatial maps corresponding to each of the grand spatial maps. In the final stage of dual-regression analysis, we tested voxel-wise for statistically significant difference between the groups using non-parametric permutation testing (10000 permutations). This results in statistical maps characterizing the group differences. These maps were thresholded at *p* < 0.005 (uncorrected) using “threshold-free cluster enhancement (TFCE)” as implemented in FSL (Smith and Nichols, 2009). The effects of age, gender, and education were statistically accounted for by including these variables as subject-wise covariates in all of the statistical models. Group comparisons were performed for (a) CN and aMCI+, (b) CN and aMCI−, (c) CN and AD, and (d) aMCI− and aMCI+.

### Gray matter morphology

To verify whether altered FC in the current study might be explained by MRI-detectable loss of gray matter (GM), a customized voxel-based morphometry (VBM) approach was implemented following the combination of the VBM toolbox version 8 (VBM8 version 435)^2^ and the Diffeomorphic Anatomical Registration through Exponentiated Lie algebra toolbox (DARTEL) (Ashburner, 2007) using SPM8 software package (Wellcome Trust Center for Neuroimaging, London)^3^ using default parameters. The native structural T1 volumes were segmented into GM, white matter (WM), and cerebrospinal fluid tissue classifications, which were then used for partial volume estimation to facilitate accurate segmentation (Tohka et al., 2004). Two denoising methods were applied: (1) a spatially adaptive non-local means denoising filter which removes noise while preserving edges and (2) a Markov Random Field denoising filter which removes isolated voxels that are unlikely to be a member of a certain tissue class (Manjon et al., 2010). The filtered segmented data were affine registered to the tissue probability maps provided by the VBM8 toolbox and then were used in DARTEL to create a study-specific customized template. The warped GM and WM segments were modulated by multiplying voxel values in the segmented images by the Jacobian determinants derived from the spatial normalization step to correct for local differences in shape. The resulting normalized modulated non-linear GM images were smoothed with an 8-mm FWHM and were used for between-group comparisons. Based on our hypothesis, we restricted the VBM analysis to only differences in GM. Total intracranial volume was not used as a covariate as the non-linear images represent volume of GM corrected for individual brain sizes. Age, gender, and education were included in the statistical model as nuisance covariates.

The resulting set of *T* values constituted the SPM(T) map. The voxel-wise results were initially displayed at *p* less than 0.005 (uncorrected) to illustrate patterns. Then, we applied *p* less than 0.001 (two-tailed, uncorrected for multiple comparisons) as a significance height threshold at a voxel-level across the GM. Given the relatively small sample size, the significance threshold was set at *p* less than 0.001 (uncorrected) in order to avoid the unintended overlook of novel findings by too conservative a threshold. The MNI coordinates were automatically calculated in SPM8 and transformed into Talairach and Tournoux (Talairach and Tournoux, 1988) by the mni2tal program^4^.

### Non-imaging statistics

All other statistical analyses were performed using Statistical Package for the Social Sciences 18.0 (SPSS, SPSS Inc., Chicago, IL, USA). For continuous measures, differences between groups were assessed using one-way ANOVA with *post hoc* Bonferroni tests to correct for multiple comparisons. Fisher’s exact test was used to compare frequency distributions of gender.

## Results

### Demographics, cognitive performance, and amyloid burden

Twelve CN, 10 aMCI−, 10 aMCI+, and 11 AD patients were compared on their demographic characteristics and neuropsychological performance scores (Table 1). Mean age of all subjects was 69.4 (SEM = 1.07) and their mean years of education was 9.7 (SEM = 0.65), of which 81% of the subjects were female. CN, aMCI−, and aMCI+ groups did not differ in their age. However, CN was older than AD. All groups did not differ in their years of education or gender distribution. As expected, CN performed significantly better on the MMSE compared to aMCI−, aMCI+, and AD groups. In addition, aMCI− and aMCI+ groups performed significantly better than AD on the MMSE.

**Table 1:** **Demographic, neuropsychological, gray matter volumetric, and cerebral amyloid burden characteristics**.

	CN	aMCI−	aMCI+	AD	*p*-value
Demographics
*n* (total *N* = 43)	12	10	10	11	
Age, years (SEM)	71.75(1.21)	70.70(1.71)	71.20(2.50)	64.18(2.41)*	0.032
Sex (% female)	75	80	80	91	0.874
Education, years (SEM)	10.33(1.23)	9.00(1.50)	10.50(1.32)	9.09(1.32)	0.790
MMSE, raw (SEM)	27.40(0.45)	23.70(0.87)*	23.40(0.79)*	18.09(1.18)*^,§,I^	<0.001
Neuropsychological tests [T-scores (SD)]
Category fluency^a^	58.80(13.42)	42.15(9.8)*	47.17(9.33)	37.07(12.86)*	0.001
Boston naming test^a^	54.95(13.01)	45.22(10.44)	46.04(9.90)	49.29(7.79)	0.117
Immediate word recall^a^	58.51(11.97)	39.49(9.85)*	36.39(9.80)*	31.23(9.46)*	<0.001
Visual construction^a^	55.14(6.89)	46.81(8.62)	50.69(11.91)	43.26(15.90)	0.134
Delayed word recall	53.03(8.55)	37.26(15.11)*	38.26(7.06)*	26.57(6.26)*	<0.001
Word recognition^a^	51.05(6.77)	40.52(10.31)	43.10(13.06)	25.95(15.42)*^,I^	<0.001
Delayed visual memory^a^	56.88(13.17)	36.83(10.59)*	37.97(10.65)*	34.75(9.53)*	<0.001
Stroop: color–word^a^	42.60(12.39)	43.07(14.12)	40.99(9.35)	24.46(7.63)*^,§,I^	0.001
PiB cortical retention [Mean (SD)]	1.051(0.077)	1.070(0.074)	1.542(0.241)*^,§^	1.692(0.228)*^,§^	<0.001

*Data are presented as means (standard error of the mean, SEM; standard deviation, SD). If analysis of variance was *p* < 0.05, a *post hoc* Bonferroni test was performed*.

*CN, cognitively normal healthy controls; aMCI−, amnestic-type mild cognitive impairment PiB-negative; aMCI+, amnestic-type mild cognitive impairment PiB-positive; AD, Alzheimer’s disease: MMSE, Mini-Mental Status Examination*.

*^a^Missing data of one to three subjects*.

*p < 0.05 (Bonferroni-corrected) compared with CN.

*^§^*p* < 0.05 (Bonferroni-corrected) compared with aMCI− patients*.

*^I^*p* < 0.05 (Bonferroni-corrected) compared with aMCI+ patients*.

In terms of other neuropsychological performances, four groups did not differ on the Boston Naming Test and a task of visual construction. Performances of aMCI−, aMCI+, and AD on word recall tasks (both immediate and delayed) as well as delayed visual memory task did not significantly differ from each other; however, they performed significantly worse than CN. On a recognition task, CN, aMCI−, and aMCI+ did not differ from each other; however, AD performed significantly worse than CN and aMCI+. On a task of response inhibition, CN, aMCI−, and aMCI+ performed similarly except for AD who performed significantly worse compared to the other groups. In addition, AD and aMCI− performed worse than CN on a category fluency test. Mean cortical retention of PiB of aMCI+ and AD was significantly higher than CN and aMCI− [*F*(3, 39) = 39.41, *p* < 0.001].

### Group differences in the DMN

**Figure 1:**
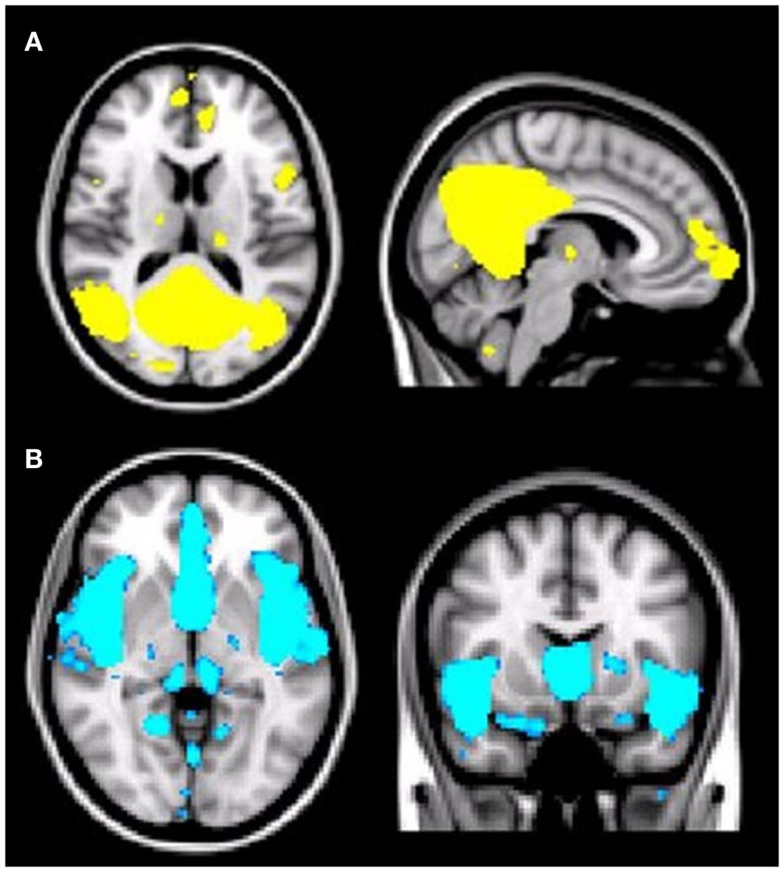
**(A)** Illustration of the default mode network (DMN) regions derived from group independent component analysis (ICA). The DMN component identified by meta-ICA analysis included the posterior cingulate cortex, precuneus, medical prefrontal cortex, lateral parietal regions, lateral temporal regions, and bilateral medial temporal regions (*p* < 0.001). **(B)** Illustration of the salience network (SN) regions derived from group independent component analysis (ICA). The SN component identified by meta-ICA analysis included the anterior cingulate cortex, presupplementary motor area, and anterior insula (*p* < 0.001).

**Figure 2:**
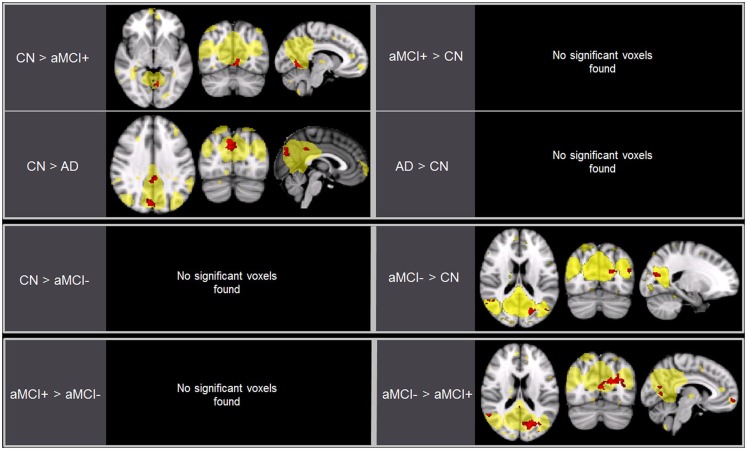
**Brain regions displaying significant differences (in red) in the default mode network (DMN) between the groups using voxel-wise comparisons (*p* < 0.005, uncorrected for multiple comparisons) masked by the overall average DMN in yellow (*p* < 0.001, uncorrected)**. Results are superimposed on the MNI-152 T1 1-mm brain template.

As predicted, ICA analysis revealed a sample-specific DMN with both the anterior and posterior regions present (Figure 1A). The results of voxel-wise between-group comparison for FC within the DMN at the threshold of *p* < 0.005 (uncorrected, TFCE) are shown in Figure 2 and Table 2. Compared to CN, aMCI+ showed decreased FC at the left lingual gyrus. There were no regions within the DMN demonstrating increased FC in aMCI+ compared to CN. Similarly, AD showed weaker DMN connectivity in the right precuneus and left posterior cingulate cortex (PCC) than CN. There were no regions showing increased DMN connectivity in AD compared to CN. In contrast, when compared to CN, aMCI− demonstrated increased DMN connectivity in the left precuneus, right superior parietal lobule, right superior temporal gyrus, left middle temporal gyrus, and left culmen. There were no regions where aMCI− showed weaker DMN connectivity compared to CN.

**Table 2:** **Regions of significant difference in functional connectivity within the default mode network between CN, aMCI−, aMCI+, and AD**.

Contrast	Brain region	BA	MNI coordinates			Extent voxels (mm^3^)	Peak *T* value
			*X*	*Y*	*Z*	
CN > aMCI+	L. lingual gyrus	18	−8	−64	2	81	4.77
CN < aMCI+	None						
CN > AD	R. precuneus	7	6	−76	36	228	5.21
	L. posterior cingulate	31	−2	−40	38	112	4.49
CN < AD	None						
CN > aMCI−	None						
CN < aMCI−	L. precuneus	31	−20	−70	16	76	4.77
	R. superior parietal lobule	7	22	−68	58	69	4.82
	R. superior temporal gyrus	22	56	−50	14	28	3.53
	L. middle temporal gyrus	39	−48	−72	18	21	4.02
	L. culmen		0	−50	−2	18	3.79
aMCI+ < aMCI−	L. cuneus	18	−16	−74	20	383	6.32
	R. superior temporal gyrus	22	54	−48	14	51	5.75
	L. superior frontal gyrus	10	−6	64	−6	47	3.67
	R. superior parietal lobule	7	28	−66	58	30	3.10
	R. inferior temporal gyrus	21	58	−8	−18	23	4.56
	L. medial frontal gyrus	10	0	64	2	17	3.27
	Culmen		0	−62	−6	11	4.16
	R. posterior cingulate	29	4	−42	16	10	3.99
aMCI+ > aMCI−	None						

*CN, cognitively normal healthy control; aMCI−, amnestic-type mild cognitive impairment PiB-negative; aMCI+, amnestic-type mild cognitive impairment PiB-positive; AD, Alzheimer’s disease; BA, Brodmann area*.

Between aMCI+ and aMCI−, aMCI− showed stronger DMN connectivity in the left cuneus, right PCC, right superior parietal lobule, right superior temporal gyrus, right inferior temporal gyrus, left superior frontal gyrus, left medial frontal gyrus, and left culmen compared to aMCI+. There were no regions demonstrating decreased DMN connectivity in aMCI− compared to aMCI+.

### Group differences in the SN

**Figure 3:**
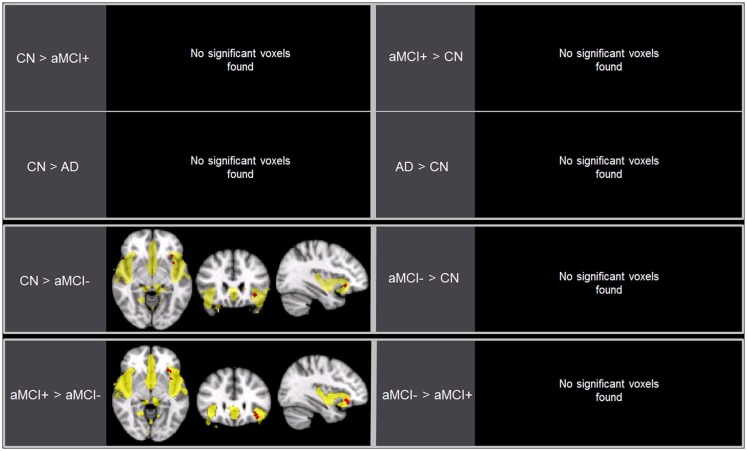
**Brain regions displaying significant differences (in red) in the salience network (SN) between the groups using voxel-wise comparisons (*p* < 0.005, uncorrected for multiple comparisons) marked by the overall average SN in yellow (*p* < 0.001, uncorrected)**. Results are superimposed on the MNI-152 T1 1-mm brain template.

Independent component analysis revealed a sample-specific SN with the anterior cingulate cortex and anterior insula (Figure 1B). The results of voxel-wise between-group comparison for FC within the SN at the threshold of *p* < 0.005 (uncorrected, TFCE) are shown in Figure 3 and Table 3. There were no differences in SN connectivity between CN, aMCI+, and AD. Compared to CN, aMCI− demonstrated decreased SN connectivity in the left inferior frontal gyrus (iFG); however, there were no regions where aMCI− showed increased SN connectivity compared to CN.

**Table 3:** **Regions of significant difference in functional connectivity within the salience network between CN, aMCI−, aMCI+, and AD**.

Contrast	Brain region	BA	MNI coordinates			Extent voxels (mm^3^)	Peak *T* value
			*X*	*Y*	*Z*	
CN > aMCI+	None						
CN < aMCI+	None						
CN > AD	None						
CN < AD	None						
CN > aMCI−	L. inferior frontal gyrus	47	−36	24	−6	16	3.99
CN < aMCI−	None						
aMCI + < aMCI−	None						
aMCI + > aMCI−	L. inferior frontal gyrus	47	−34	26	−8	55	4.95

*CN, cognitively normal healthy control; aMCI−, amnestic-type mild cognitive impairment PiB-negative; aMCI+, amnestic-type mild cognitive impairment PiB-positive; AD, Alzheimer’s disease; BA, Brodmann area*.

Between aMCI+ and aMCI−, aMCI− demonstrated decreased SN connectivity in the left iFG compared to aMCI+. There were no regions where aMCI− demonstrated stronger SN connectivity than aMCI+.

### Group differences in regional brain volume

**Figure 4:**
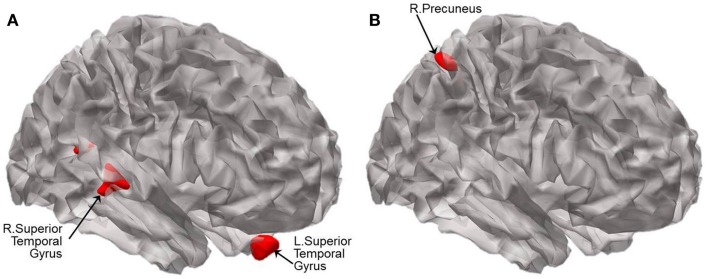
**Statistical parametric maps showing the results of analysis of covariance of VBM at *p* < 0.005 (uncorrected)**. Brain regions displaying significant differences in brain volume are shown in red between **(A)** CN and aMCI−, and between **(B)** CN and aMCI+. Results are superimposed using the cortex_20484.surf template in SPM8. The peak voxels of clusters meeting the *p* < 0.001 (uncorrected) cluster-wise criterion for the contrasts are listed in Table 4.

For illustration, the results of VBM between-group comparisons are displayed at the threshold of *p* < 0.005 (uncorrected) as shown in Figure 4. Compared to CN, aMCI− showed atrophy in the bilateral superior temporal gyri (BA 38 and 13) and left uncus whereas aMCI+ showed atrophy in the right precuneus and left lingual gyrus (Figure 4; Table 4). The peak voxels of clusters meeting the *p* < 0.001 (uncorrected) cluster-wise criterion for the contrasts are listed in Table 4.

**Table 4:** **Regions of significant difference in brain volume between CN, aMCI−, and aMCI+**.

Contrast	Brain region	BA	MNI coordinates			Extent voxels (mm^3^)	Peak *T* value
			*X*	*Y*	*Z*	
CN > aMCI+	R. precuneus	7	29	−56	56	71	5.07
	L. lingual gyrus	18	−11	−89	−20	21	4.12
CN > aMCI−	L. uncus	20	−35	−12	−35	20	4.50
	L. superior temporal gyrus	38	−33	14	−38	74	4.24
	R. superior temporal gyrus	13	47	−48	15	21	4.05

*CN, cognitively normal healthy control; aMCI−, amnestic-type mild cognitive impairment PiB-negative; aMCI+, amnestic-type mild cognitive impairment PiB-positive; BA, Brodmann area*.

## Discussion

The present study showed that DMN FC differences between CN, aMCI+, and AD occur in the expected direction consistent with the results from previous studies on the differences between CN, overall aMCI, and AD. Both AD and aMCI+ showed decreased DMN FC compared to CN. The novel aspect of the present report was the comparisons of aMCI− with CN and aMCI+. aMCI− demonstrated increased FC strength in several DMN regions, mainly involving the precuneus, posterior cingulate, superior parietal, and superior temporal regions compared to aMCI+ as well as CN. Furthermore, aMCI− showed decreased FC strength of the SN in the left iFG compared to both CN and aMCI+. The present findings show that aMCI− has quite a different functional brain connectivity compared to aMCI+ despite overall similarity in cross-sectional cognitive features. To the best of our knowledge, this is the first study to directly examine FC alterations in both DMN and SN between aMCI+ and aMCI−.

Functional connectivity alterations in the posterior regions (i.e., the precuneus and PCC) of the DMN in AD and aMCI have been repeatedly found in previous task-related as well as task-free fMRI studies (Lustig et al., 2003; Greicius et al., 2004; Celone et al., 2006). The current study extends our knowledge about the DMN FC alterations in aMCI by specifying that previously reported FC alterations are distinctive of aMCI with high Aβ burden. The most widely held hypothesis to account for such finding is that the decreased FC in the posterior association cortices is not only secondary to local AD-related neuropathological abnormalities in the very posterior DMN regions but also reflects distant effects of neuronal damage in the remote brain regions, such as the medial temporal lobe including hippocampus, based on its massive connectivity with widespread brain regions (Arnold et al., 1991; Jack et al., 2008; Bourgeat et al., 2010).

Unlike aMCI+ whose SN FC was comparable to CN, aMCI− exhibited decreased FC strength of the SN in the left iFG compared to CN and aMCI+. Furthermore, FC strength in the posterior regions of the DMN increased in aMCI− compared to CN and aMCI+. The present study does not demonstrate the causal relationship between memory difficulties and FC disruptions. However, given the role of the SN in disengaging the DMN when on a cognitive task (Rilling et al., 2008; Sridharan et al., 2008; Sharp et al., 2010; Bonnelle et al., 2012), the ability of aMCI− to disengage the DMN may be compromised resulting in manifestation of reduced memory performance from inefficient processing of information. Alternatively, increased FC of the DMN may indicate greater recruitment of the regions in order to compensate for atrophied temporal pole given that the posterior DMN has been implicated in memory retrieval (Shapira-Lichter et al., 2013).

The differential network changes of aMCI− and aMCI+ are also noteworthy when explicated in conjunction with the differential patterns of atrophy in aMCI− versus aMCI+ compared to CN. In aMCI+, atrophy was observed in the same region where decreased FC was found in AD compared to CN (i.e., right precuneus of the DMN), which is also one of the regions where Aβ aggregates very early in the AD process. In contrast to aMCI+, the regions of FC alterations and atrophy do not necessarily overlap in aMCI−. The current findings suggest that decreased FC of the SN in aMCI− may be associated, at least in part, to atrophied superior temporal gyri given that the regions share dense connection with the regions of the SN (Augustine, 1996).

The results of the present study point to the DMN as an important intrinsic network differentiating aMCI− and aMCI+ as well as from CN. Challenges remain, however, in conceptualization of the etiology behind aberrant FC in the PCC and iFG in aMCI−. The FC alterations in aMCI− likely reflect non-AD pathology, although the exact nature of the pathology is still not clearly elucidated. Potential candidate pathologies underlying the impairment include cerebrovascular disease, hippocampal sclerosis, or Lewy body disease (Bennett et al., 2005; Jicha et al., 2006; Schneider et al., 2007). Alternatively, FC alterations in aMCI− may reflect underlying dysregulated lipid metabolism. A recent study from our research group suggested that cognitive deficits and brain atrophy in aMCI− are associated with decreased serum apolipoprotein A-1 (APOA1), the major component of HDL cholesterol independently of Aβ and vascular burden; the authors posited that antioxidant and/or anti-inflammatory properties of APOA1 as well as its critical role in reverse cholesterol transport may account for the contribution of the lipoprotein to non-AD brain damage (Choi et al., 2013). Additional etiological considerations for aberrant FC of the SN and the DMN may include mood disorders. A recent study on major depressive disorder found decreased FC within the SN as well as aberrant inter-network FC between subsystems of the DMN and the central executive network in patients compared to healthy adults (Manoliu et al., 2013). Future studies are needed to examine and verify the relationship between non-AD pathological contributions and altered FC of the DMN and SN in aMCI− compared to aMCI+ and CN in order to elucidate the etiology.

This study has some limitations. First, the sample size in each group is relatively small. We tried to overcome statistical challenges due to small sample size by utilizing dual-regression with permutations; also, by combining all subjects in our gICA instead of using just CN, we ended up with more conservative results when describing alterations in FC in participants. Second, all presented data are cross-sectional in nature. Longitudinal changes in the networks may enable in-depth investigation of properties of alterations in these networks.

In conclusion, our results indicate that despite very similar cross-sectional profile of cognitive deficits, aMCI individuals with very low Aβ burden have quite different connectivity alteration pattern in the DMN and SN, compared to those with high Aβ burden. From a practical point of view, this discrepancy in the patterns of FC changes between the two aMCI groups may be utilized as a cost-effective biomarker for differentiating those who are at higher risk for AD dementia from those who are related to non-AD pathological processes among clinically defined overall aMCI individuals.

## Author Contributions

DY made contribution to the design of the work and performed the data analyses and prepared the manuscript. YC, MB, BS, ES, JH, and JP made substantial contribution to the acquisition of the data and provided critical intellectual reviews. DL provided a substantial contribution to the design of the work as well as to the analyses and interpretation of data; and, he critically revised the paper and approved the final version. JW made contribution to the conception of the work and acquisition of the data. All authors approved the final version to be published.

## Conflict of Interest Statement

The authors declare that the research was conducted in the absence of any commercial or financial relationships that could be construed as a potential conflict of interest.

## Footnotes

^1^www.fmrib.ox.ac.uk/fsl

^2^http://dbm.neuro.uni-jena.de/vbm/

^3^http://www.fil.ion.ucl.ac.uk/spm/software/spm8/

^4^http://imaging.mrc-cbu.cam.ac.uk/downloads/MNI2tal/
